# Impact of per- and polyfluoroalkyl substances on diabetic kidney disease

**DOI:** 10.3389/fendo.2025.1594897

**Published:** 2025-09-09

**Authors:** Siyuan Song, Liji Huang, Xiqiao Zhou, Yuan Han, Jiangyi Yu

**Affiliations:** ^1^ Department of Endocrinology, Jiangsu Province Hospital of Chinese Medicine, Affiliated Hospital of Nanjing University of Chinese Medicine, Nanjing, China; ^2^ Department of Nephrology, Children’s Hospital of Nanjing Medical University, Nanjing, China

**Keywords:** per-and polyfluoroalkyl substances, network toxicology, diabetic, molecular docking, diabetic kidney disease

## Abstract

**Purpose:**

This study aims to elucidate the mechanistic role of Per- and Polyfluoroalkyl Substances (PFAS) in the pathogenesis and progression of diabetic kidney disease (DKD).

**Methods:**

This study systematically evaluated the toxicity profiles of PFAS compounds utilizing PubChem, ProTox 3.0, and ChEMBL databases. Potential PFAS-related targets were predicted through SwissTargetPrediction and SuperPred platforms. Gene targets associated with DKD were compiled from the GeneCards and OMIM databases. Intersection analysis of PFAS and DKD-related targets was performed to identify candidate genes. A protein-protein interaction network was constructed using STRING to delineate hub targets. Functional enrichment analyses were subsequently conducted via DAVID to elucidate underlying biological processes and pathways. Validation of hub targets encompassed immunohistochemical staining, single-cell expression profiling, subcellular localization assays, and gene expression analyses using external datasets from the Human Protein Atlas (HPA) and Gene Expression Omnibus (GEO). Furthermore, correlations between immune cell infiltration and gene set enrichment analysis (GSEA) were performed to investigate potential mechanistic links. Finally, molecular docking simulations of PFAS compounds with hub proteins were executed using Discovery Studio and CDOCKER to predict binding interactions.

**Results:**

A total of 424 PFAS-associated targets were identified, alongside 9,999 potential toxic targets related to DKD. KEGG pathway enrichment analysis revealed that PFAS toxicity in DKD is implicated in critical signaling pathways, including nitrogen metabolism, peroxisome proliferator-activated receptor (PPAR) signaling, endocrine resistance, insulin resistance, and AMP-activated protein kinase (AMPK) signaling. Hub targets identified comprised MMP9, BCL2, CYP3A43, ACE, HNF4A, HSP90AA1, AGTR1, MMP2, AGTR2, and HMGCR. GSEA further indicated that these hub targets may contribute to immune-mediated renal injury. Molecular docking simulations substantiated strong binding affinities between PFAS compounds and the identified hub proteins, supporting their potential mechanistic involvement.

**Conclusion:**

This study provides a theoretical framework for elucidating the toxic targets and underlying mechanisms through which PFAS contribute to the pathogenesis of DKD.

## 1 Introduction

Per- and Polyfluoroalkyl Substances (PFAS) are a class of synthetic organic compounds characterized by the substitution of all or part of the hydrogen atoms on the carbon chain with fluorine atoms, forming highly stable C–F covalent bonds (1). Owing to the exceptionally high bond energy of the carbon–fluorine (C–F) bond, PFAS demonstrate remarkable thermal and chemical stability, as well as pronounced resistance to hydrolysis, photolysis, and biodegradation. These unique physicochemical properties have facilitated their extensive use across a wide range of industrial applications, including textiles, chemical manufacturing, electronics, and electroplating (2). However, PFAS can be released into the environment during production, processing, and daily use, resulting in their persistence in various environmental media and biological systems (3). Due to their environmental persistence and resistance to degradation, PFAS have become a pressing global concern as pollutants with significant implications for public health. Recent toxicological investigations have demonstrated that PFAS can bioaccumulate and biomagnify along the food chain in various organisms, including fish and mammals, thereby exerting detrimental toxic effects (4).

Since the 1990s, China’s rapid industrial expansion has been accompanied by significant growth in the fluorochemical industry, resulting in a marked increase in the production and consumption of fluorinated compounds. The Bohai Sea, China’s largest semi-enclosed inland sea, is characterized by a unique natural ecosystem and holds considerable geopolitical and strategic significance. It plays a vital role in supporting the socio-economic development of Beijing, Tianjin, and the broader Bohai Rim region. The Bohai Sea is also the confluence of more than 40 rivers, including major waterways such as the Haihe, Yellow, and Liao Rivers (5). The continuous discharge of substantial volumes of industrial wastewater and domestic sewage from fluorochemical industrial parks in the Bohai region has resulted in widespread PFAS contamination in adjacent inland rivers, atmospheric environments, and surface waters (6). Once introduced into the marine environment, a portion of PFAS compounds adsorb to sediments and bioaccumulate in marine organisms through direct filter-feeding, ultimately posing risks to human health.

Diabetic kidney disease (DKD) is among the most prevalent and severe complications of diabetes mellitus (DM), significantly contributing to increased morbidity and mortality in affected patients (7). Currently, over 400 million people worldwide are affected by diabetes, and this number is projected to rise to 600 million by 2035 and further to 700 million by 2045 (8). Among individuals with DM, approximately 20% are expected to develop DKD (9). Data from the National Health and Nutrition Examination Survey (NHANES) have demonstrated that individuals with elevated PFAS concentrations exhibit significantly reduced estimated glomerular filtration rates (eGFR) (10). While several studies have indicated that chronic exposure to PFAS may elevate the risk of developing DKD (11), the precise molecular mechanisms driving this association remain poorly understood.

Traditional toxicological approaches encounter considerable challenges in evaluating the complex biological impacts of emerging environmental pollutants, particularly regarding the timeliness and comprehensiveness of exposure assessment (12). Conventional toxicity assessments predominantly concentrate on exposure metrics and often evaluate the isolated effects of one or a limited number of molecular targets, frequently neglecting the broader systemic impacts and intricate toxicological pathways involved. In contrast, environmental pollutants commonly perturb multiple signaling targets across diverse biological systems, disrupting complex molecular networks and ultimately eliciting multifaceted toxic responses (13). Therefore, the development of rapid and robust evaluation methods to assess the toxicity of emerging environmental contaminants is imperative. Network toxicology, an approach derived from network pharmacology and grounded in systems biology, leverages network-based analyses to comprehensively characterize the interactions within biological systems (14). It utilizes the design of multi-target drug molecules based on selected signaling molecules and integrates bioinformatics with high-throughput omics data to identify active compounds or design novel therapeutic agents. As such, it represents a new interdisciplinary approach combining pharmacology and information technology (15). The fundamental principle of network toxicology involves predicting the toxicological targets of environmental pollutants based on their chemical structures through the integration of multiple databases. This is followed by comprehensive functional and signaling pathway analyses of gene targets obtained from genomic repositories. Ultimately, this approach enables the construction of an interactive network that delineates the relationships among toxicity, toxic chemical components, molecular targets, and associated effect pathways (16).

Therefore, this study adopts a network toxicology approach that integrates computational modeling of toxicological pathways with mechanistic network predictions to identify and analyze potential toxic targets (17). This strategy is applied to elucidate the nephrotoxic pathways implicated in exposure-related DKD, with the objective of characterizing the toxicological properties of PFAS and predicting their associated toxicity and molecular mechanisms. Ultimately, this work aims to provide valuable insights into efficient methods for assessing environmental pollutant toxicity and to establish a foundational framework for investigating diseases linked to such exposures.

## 2 Method

### 2.1 Toxicity assessment of PFAS compounds

The SMILES representations for twenty principal PFAS compounds (**Table 1**) were obtained from the PubChem repository (https://pubchem.ncbi.nlm.nih.gov/) (18). These molecular identifiers were subsequently analyzed using ProTox version 3.0 (https://tox.charite.de/protox3/) (19) and the ChEMBL database (https://www.ebi.ac.uk/chembl/) (20) to enable comprehensive computational assessment of their toxicological profiles.

**Table 1:** Molecular formula, molecular weight and SMILES structure of PFAS.

Name	Molecular formulas	Molecular weights (g/mol)	SMILES structures
PFOA	C8HF15O2	414.07	C(=O)(C(C(C(C(C(C(C(F)(F)F)(F)F)(F)F)(F)F)(F)F)(F)F)(F)F)O
PFHxA	C6HF11O2	314.05	C(=O)(C(C(C(C(C(F)(F)F)(F)F)(F)F)(F)F)(F)F)O
PFHxS	C6HF13O3S	400.12	C(C(C(C(F)(F)S(=O)(=O)O)(F)F)(F)F)(C(C(F)(F)F)(F)F)(F)F
PFBA	C4HF7O2	214.04	C(=O)(C(C(C(F)(F)F)(F)F)(F)F)O
FTOH	C10H5F17O4S	544.18	C(COS(=O)(=O)O)C(C(C(C(C(C(C(C(F)(F)F)(F)F)(F)F)(F)F)(F)F)(F)F)(F)F)(F)F
FTS	C22H30O2S	358.5	CC(=CCCC(=CCCC(=CCSC1=CC=CC=C1C(=O)O)C)C)C
PFPeA	C5HF9O2	264.05	C(=O)(C(C(C(C(F)(F)F)(F)F)(F)F)(F)F)O
PFHpA	C7HF13O2	364.06	C(=O)(C(C(C(C(C(C(F)(F)F)(F)F)(F)F)(F)F)(F)F)(F)F)O
PFNA	C9HF17O2	464.08	C(=O)(C(C(C(C(C(C(C(C(F)(F)F)(F)F)(F)F)(F)F)(F)F)(F)F)(F)F)(F)F)O
PFDA	C10HF19O2	514.08	C(=O)(C(C(C(C(C(C(C(C(C(F)(F)F)(F)F)(F)F)(F)F)(F)F)(F)F)(F)F)(F)F)(F)F)O
6:2 FTSA	C8H5F13O3S	428.17	C(CS(=O)(=O)O)C(C(C(C(C(C(F)(F)F)(F)F)(F)F)(F)F)(F)F)(F)F
PFPrA	C3HF5O2	164.03	C(=O)(C(C(F)(F)F)(F)F)O
GenX	C6HF11O3	330.05	C(=O)(C(C(F)(F)F)(OC(C(C(F)(F)F)(F)F)(F)F)F)O
FTOH	C10H5F17O4S	544.18	C(COS(=O)(=O)O)C(C(C(C(C(C(C(C(F)(F)F)(F)F)(F)F)(F)F)(F)F)(F)F)(F)F)(F)F
PFOSA	C8H2F17NO2S	499.15	C(C(C(C(C(F)(F)S(=O)(=O)N)(F)F)(F)F)(F)F)(C(C(C(F)(F)F)(F)F)(F)F)(F)F
ADONA	C7H2F12O4	378.07	C(C(C(=O)O)(F)F)(OC(C(C(OC(F)(F)F)(F)F)(F)F)(F)F)F
NMeFOSA	C9H4F17NO2S	513.169	CNS(=O)(=O)C(C(C(C(C(C(C(C(F)(F)F)(F)F)(F)F)(F)F)(F)F)(F)F)(F)F)(F)F
NEtFOSA	C10H6F17NO2S	527.2	CCNS(=O)(=O)C(C(C(C(C(C(C(C(F)(F)F)(F)F)(F)F)(F)F)(F)F)(F)F)(F)F)(F)F
6:2 FTOH	C8H5F13O	364.1	C(CO)C(C(C(C(C(C(F)(F)F)(F)F)(F)F)(F)F)(F)F)(F)F
6:2 FTSAAm	C13H18F13N2O2S+	513.34	C[NH+](C)CCCNS(=O)(=O)CCC(C(C(C(C(C(F)(F)F)(F)F)(F)F)(F)F)(F)F)(F)F

### 2.2 Identification of PFAS-associated molecular targets

The selected PFAS compounds, represented in SMILES format and sourced from the PubChem database, were analyzed using the SwissTargetPrediction platform (http://www.swisstargetprediction.ch/)(21) and the SuperPred tool (https://prediction.charite.de) (22). In both systems, *Homo sapiens* was designated as the target organism to ensure biological relevance to humans, with a screening threshold set at “Probability ≥ 0.” SwissTargetPrediction utilizes machine learning algorithms that analyze molecular fingerprints alongside curated ligand–protein interaction datasets to predict probable targets. Likelihood scores are assigned based on structural and chemical feature comparisons. To maximize the inclusivity of potential targets, a minimum probability cutoff of zero was applied, thereby prioritizing sensitivity in target identification (23). Subsequently, target specificity was enhanced through systematic refinement and enrichment processes. These computational tools collectively enabled the prediction, filtration, and validation of molecular targets potentially interacting with PFAS compounds.

### 2.3 Identification of DKD-associated targets

To uncover genes implicated in DKD, searches were conducted in the GeneCards database (https://www.genecards.org/) (24) and the Online Mendelian Inheritance in Man (OMIM) resource (https://omim.org/) (25) using the keyword “diabetic kidney disease.” After removing duplicate entries, a comprehensive set of DKD-related targets was compiled by integrating data from both databases. To enhance dataset relevance, the median “Relevance score” across all identified targets was used as a threshold for filtering (26). Employing the median as a cutoff ensures uniformity and facilitates the integration of heterogeneous gene expression datasets. This strategy strikes a balance between selectivity and biological significance, while mitigating potential biases arising from variable data volumes across platforms (27). Additionally, Venn diagram analysis was performed to identify overlapping targets shared between PFAS-associated and DKD-related gene sets. These intersecting targets were designated as candidate toxicological targets through which PFAS may contribute to the pathogenesis of DKD.

### 2.4 Construction of protein-protein interaction network and identification of hub targets

The intersecting targets identified as potential mediators of PFAS-induced DKD-represented by the overlapping region of the Venn diagram-were submitted to the STRING database (https://string-db.org/) using the “Multiple Proteins” analysis function. The species parameter was set to *Homo sapiens*, and the minimum required interaction score was configured to “high confidence” (>0.7). Additionally, the false discovery rate (FDR) threshold was set to a stringent level to minimize false positives. Applying a high confidence cutoff (>0.7) effectively excludes low-quality or speculative interactions, retaining only experimentally validated or strongly supported associations (28, 29). Concurrently, stringent FDR control significantly reduces the likelihood of false-positive signals arising from random errors, thereby enhancing the reliability and reproducibility of the interaction network.

The results obtained from the STRING database were imported into Cytoscape software (version 3.10.3) to calculate network parameters for each node and to optimize the visualization of molecular interactions (30). A PPI network was constructed based on the topological characteristics of the nodes. Using the CytoNCA plugin “Centiscape”, key topological parameters for each gene node were calculated, including Closeness Centrality, Betweenness Centrality, and Degree Value. Hub targets were identified based on the following three criteria:

1. Closeness centrality > average value;
2. Betweenness centrality > average value;
3. Degree value > average value.

The selection of these three parameters reflects their complementary roles in network topology analysis: degree centrality quantifies the importance of a node based on the number of its direct connections, thereby identifying locally dense hub nodes (31); betweenness centrality measures the extent to which a node functions as a critical intermediary facilitating global information flow within the network (32); and closeness centrality evaluates the ability of a node to efficiently disseminate information across the entire network (33). The combined application of these metrics enables a more comprehensive and nuanced assessment of node significance. By integrating multiple topological indicators, this approach minimizes selection bias and enhances the robustness and reliability of the identified hub targets (34).

### 2.5 Functional enrichment analysis

To elucidate the biological functions of potential targets involved in PFAS-induced DKD, Gene Ontology (GO) and Kyoto Encyclopedia of Genes and Genomes (KEGG) pathway enrichment analyses were performed using the DAVID database (https://david.ncifcrf.gov). The GO analysis encompassed three domains: biological processes (BP), cellular components (CC), and molecular functions (MF), providing a comprehensive overview of the primary biological roles of these targets. KEGG enrichment analysis identified key signaling pathways associated with PFAS-induced DKD targets, applying a FDR threshold of < 0.05 to ensure statistical robustness. Furthermore, KEGG pathway enrichment was specifically conducted on the subset of selected hub targets to delineate their involvement in DKD-related pathways, thereby underscoring critical signaling cascades implicated in disease pathogenesis.

### 2.6 Validation of the hub targets in the different databases

To validate the expression patterns of the identified hub targets, immunohistochemical (IHC) staining data were retrieved from the Human Protein Atlas (HPA; https://www.proteinatlas.org/). Protein expression levels were evaluated across various organs by examining staining intensity, the identity of positively stained cell types, and tissue-specific distribution. Additionally, single-cell transcriptomic analyses were conducted to characterize target gene expression across distinct cell populations, including endocrine and epithelial cells. Uniform Manifold Approximation and Projection (UMAP) was employed to visualize gene expression distribution among heterogeneous cell types. Subcellular localization of the corresponding proteins was further assessed using fluorescence microscopy, providing spatial resolution of protein enrichment within key cellular compartments such as the nucleus, cytoplasm, mitochondria, and endoplasmic reticulum.

Differential expression of hub genes between normal renal tissues and those affected by chronic kidney disease (CKD) was validated using the Nephroseq database (https://www.nephroseq.org/). To corroborate these findings in the context of DKD, gene expression datasets were obtained from the Gene Expression Omnibus (GEO; https://www.ncbi.nlm.nih.gov/geo/), with particular focus on the GSE30122 dataset comprising 19 DKD samples and 50 normal controls, enabling assessment of differential gene expression between diseased and healthy renal tissues.

### 2.7 Correlation of immune cell infiltration

To explore the relationships among immune cells during infiltration, the immune microenvironment was profiled using CIBERSORT (35), enabling comparison of relative immune cell subsets and immune scores in the GSE30122 dataset. A significance threshold of *P* < 0.05 was applied for selecting features for further analysis. Correlation matrices of immune infiltration were then generated, categorizing correlation strength as weak (absolute coefficients between 0.10 and 0.39), moderate (0.40 to 0.69), and strong (ranging from 0.70 to 0.89).

### 2.8 GSEA

GSEA was performed using GSEA software (version 3.0), obtained from the Broad Institute website (http://software.broadinstitute.org/gsea/index.jsp) (36). Based on the expression levels of core genes, samples were stratified into high-expression (≥50%) and low-expression (<50%) groups. Curated gene sets (c2.cp.kegg.v7.4.symbols.gmt) were sourced from the Molecular Signatures Database (MSigDB, http://www.gsea-msigdb.org/gsea/downloads.jsp) (37) for pathway and molecular mechanism analyses. The minimum and maximum gene set sizes were set to 5 and 5000, respectively, with 1,000 permutations performed to ensure robust statistical assessment. Pathways achieving a nominal *P* < 0.05 and a FDR < 0.25 were considered statistically significant.

### 2.9 Molecular docking

Discovery Studio, a specialized software for molecular simulations in the life sciences, is commonly employed in drug development and biomacromolecular modeling, particularly for analyzing protein and antibody structures (38). In this study, PFAS compounds were employed as ligands for molecular docking simulations. Receptor proteins were selected based on hub targets exhibiting the highest degree centrality within the PPI network. Three-dimensional structures of the PFAS molecules were obtained from the PubChem database in SDF format. Corresponding 3D protein structures of the primary toxic targets were retrieved from the RCSB Protein Data Bank (http://www1.rcsb.org/), specifying Homo sapiens as the source organism and restricting resolution to 0–3.0 Å. Using Discovery Studio 2019, receptor structures were preprocessed by adding hydrogen atoms, removing crystallographic water molecules, and reconstructing missing loop regions. Potential binding pockets were subsequently predicted. Ligands were prepared through hydrogenation and conformational analysis. Molecular docking simulations were performed with the CDOCKER module to predict the optimal binding orientations between PFAS ligands and selected targets (39). CDOCKER interaction energies were calculated, with higher scores indicative of stronger ligand–receptor binding affinities.

## 3 Results

### 3.1 Target screening for PFAS-induced DKD

**Figure 1:**
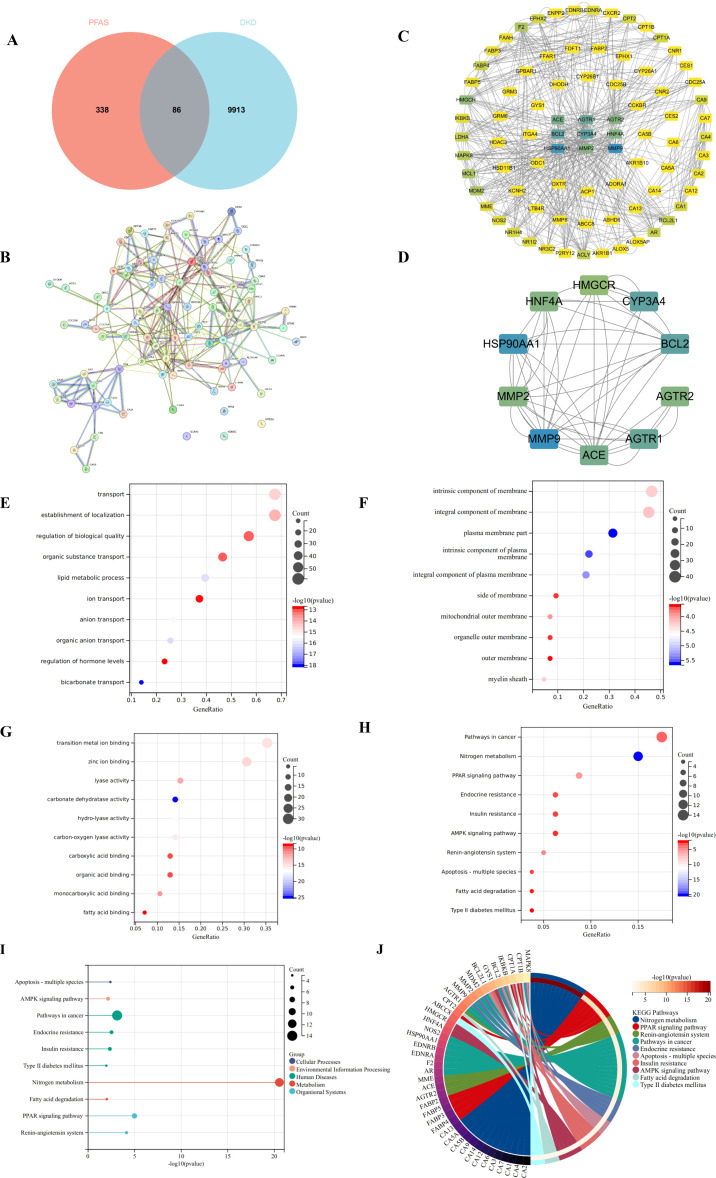
Target screening for PFAS-induced DKD and functional enrichment analysis. **(A)** Venn diagram of PFAS targets and DKD-related targets; Red represents PFAS-related targets, and blue represents DKD-related targets. **(B)** PPI network of the intersection targets through STRING database; **(C)** PPI Network of the intersection targets through Cytoscape 3.9.0; Darker blue nodes indicate higher degree in the PPI network. **(D)** Top 10 hub targets through CytoHubba plugin; **(E)** Biological process analysis of hub targets; **(F)** Cellular Components analysis of hub targets; **(G)** Molecular function analysis of hub targets; **(H)** KEGG analysis of hub targets; The size of each bubble represents the number of enriched genes (Count), while the color gradient from light to deep blue indicates increasing −log_10_(P value), with darker colors representing higher enrichment significance. **(I)** Lollipop Plot of KEGG analysis; The size of each bubble represents the number of enriched genes (Count), while the horizontal axis shows −log_10_(P value), where larger values denote more significant enrichment. **(J)** Circular plot of KEGG analysis; Each color represents a different pathway, and the shift toward yellow reflects an increase in −log_10_(P value), indicating stronger enrichment.

Targets for 20 major PFAS compounds were predicted using the SwissTargetPrediction and SuperPred databases. Following data integration and removal of duplicates, a total of 424 unique PFAS-related targets were identified. Gene targets associated with DKD were obtained from the GeneCards and OMIM databases, resulting in 9,999 unique targets after filtering. Intersection analysis using Venny 2.1.0 revealed 86 overlapping targets (**Figure 1A**), which represent potential toxicological mediators through which PFAS may contribute to the pathogenesis of DKD.

### 3.2 Construction of PPI network and identification of hub targets

To analyze these common targets, a PPI network was constructed by submitting the 86 shared targets to the STRING database (**Figure 1B**), with parameters set to “Multiple proteins,” species designated as *Homo sapiens*, and a high-confidence minimum interaction score. This yielded a network comprising 83 nodes and 572 edges (**Figure 1C**), illustrating the intricate interactions between PFAS-associated toxic targets and the pathophysiology of DKD. To further elucidate the mechanisms underlying PFAS-induced toxicity in DKD, the network was imported into Cytoscape 3.9.0, where the CytoHubba plugin was employed for clustering analysis. The top 10 hub targets, identified based on degree centrality, included MMP9, BCL2, CYP3A43, ACE, HNF4A, HSP90AA1, AGTR1, MMP2, AGTR2, and HMGCR (**Figure 1D**), suggesting their pivotal roles in mediating PFAS-induced DKD toxicity.

### 3.3 Functional enrichment analysis

GO and KEGG pathway enrichment analyses were conducted on the 86 candidate targets associated with PFAS-induced DKD toxicity. GO analysis revealed that, in terms of BP, these targets are primarily involved in transport, regulation of biological processes, and lipid metabolic processes (**Figure 1E**). Regarding CC, the targets are predominantly associated with intrinsic components of membranes and plasma membrane parts (**Figure 1F**). For MF, the targets are mainly linked to transition metal ion binding, zinc ion binding, and lyase activity (**Figure 1G**). KEGG pathway enrichment identified the top 10 significantly enriched pathways, with the most prominent including nitrogen metabolism, PPAR signaling, endocrine resistance, insulin resistance, and AMPK signaling pathways (**Figure 1H**). Notably, the majority of these pathways are related to metabolic regulation (**Figures 1I, J**). Collectively, these findings provide valuable insights into the potential mechanisms by which PFAS contribute to DKD toxicity, emphasizing key biological processes and signaling pathways involved in disease progression.

### 3.4 Validation of the hub targets in the different databases

**Figure 2:**
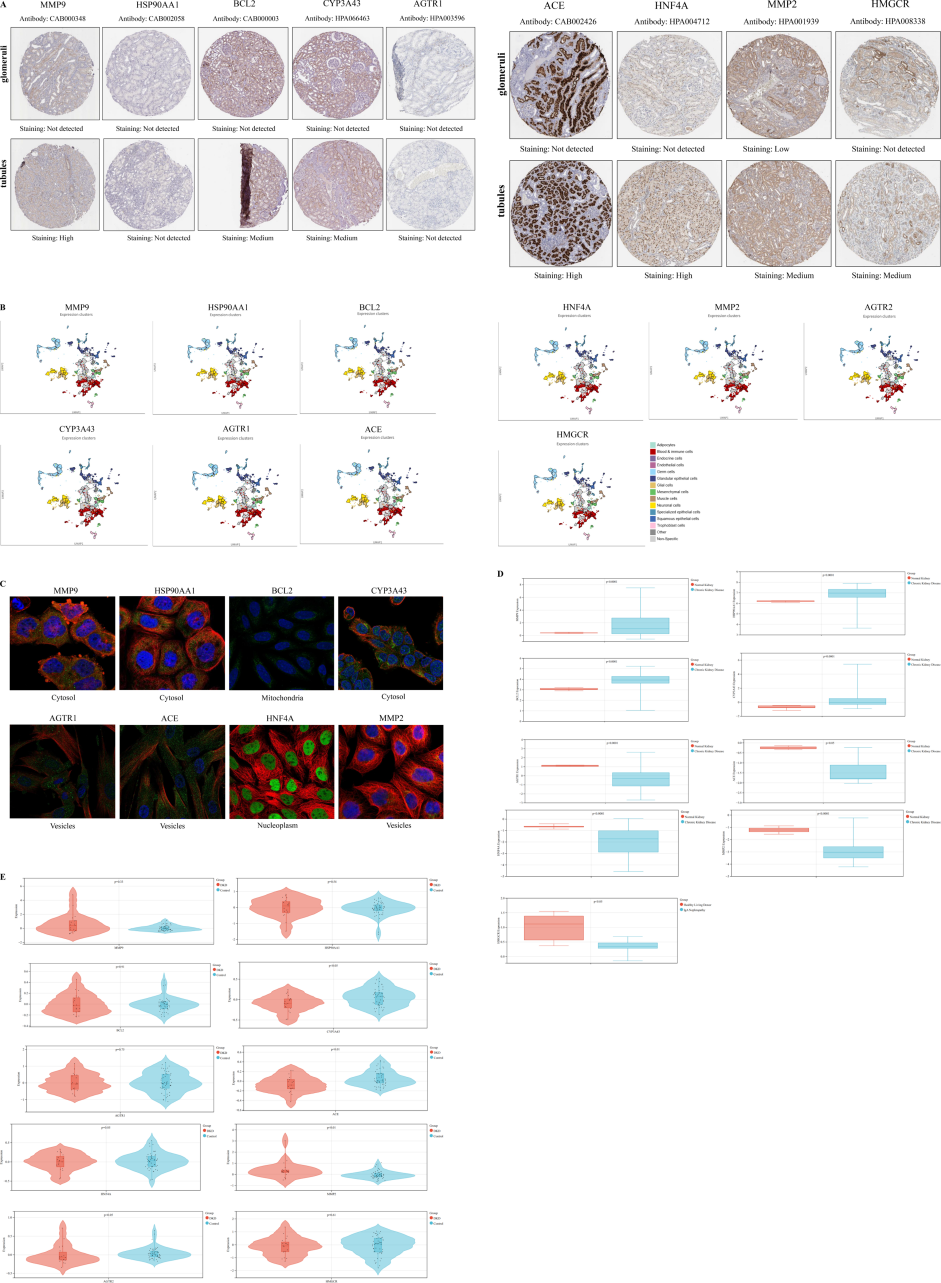
Validation of the hub targets in the different databases. **(A)** Immunohistochemical staining images depicting the expression of hub targets in renal tubules and glomeruli in the HPA database; **(B)** Single-cell maps illustrating the distribution of hub targets across different cell types in the HPA database; **(C)** Subcellular localization analysis of hub targets in the HPA database; **(D)** Boxplots depicting the differential expression of hub targets between normal kidney and those affected by chronic kidney disease in the Nephroseq database; Red represents normal kidney, while blue represents chronic kidney disease. A *P* value less than 0.05 was considered indicative of statistical significance. **(E)** Violin plots depicting the differential expression of hub targets between DKD and Control in the GSE30122 dataset; Red represents the DKD, while blue represents the Control. A *P* value less than 0.05 was considered indicative of statistical significance.

Validation using the HPA database revealed that the hub targets implicated in PFAS-induced DKD toxicity-namely MMP9, BCL2, CYP3A43, ACE, HNF4A, and HMGCR-were predominantly localized in renal tubules, with no detectable expression in glomeruli. HSP90AA1 and AGTR1 were undetected in both tubules and glomeruli. MMP2 demonstrated moderate expression in the renal tubules but was expressed at low levels in the glomeruli. Immunohistochemical data for AGTR2 are currently unavailable in the HPA database (**Figure 2A**). Single-cell transcriptomic analysis indicated the expression of these targets across germ cells, neuronal cells, blood immune cells, and endocrine cells (**Figure 2B**). Subcellular localization analysis further showed that HSP90AA1, MMP9, and CYP3A43 were primarily localized in the cytosol; BCL2 was enriched in mitochondria; AGTR1, ACE, and MMP2 were predominantly found in vesicles; and HNF4A was localized within the nucleoplasm (**Figure 2C**).

Analysis of the Nephroseq database revealed that MMP9, HSP90AA1, BCL2, and CYP3A43 were significantly upregulated in CKD, whereas AGTR1, ACE, HNF4A, MMP2, and HMGCR exhibited significant downregulation, with all differences reaching statistical significance (*P* < 0.05). Expression data for AGTR2 were not available in this database (**Figure 2D**). In the GSE30122 dataset, several genes-including MMP9, HSP90AA1, BCL2, MMP2, AGTR1, and HMGCR-were upregulated in DKD, whereas ACE, HNF4A, CYP3A43, and AGTR2 were downregulated. Among these, the differential expression of CYP3A43, ACE, MMP2, and AGTR2 was statistically significant (*P* < 0.05) (**Figure 2E**).

### 3.5 Correlation of immune cell infiltration and GSEA

**Figure 3:**
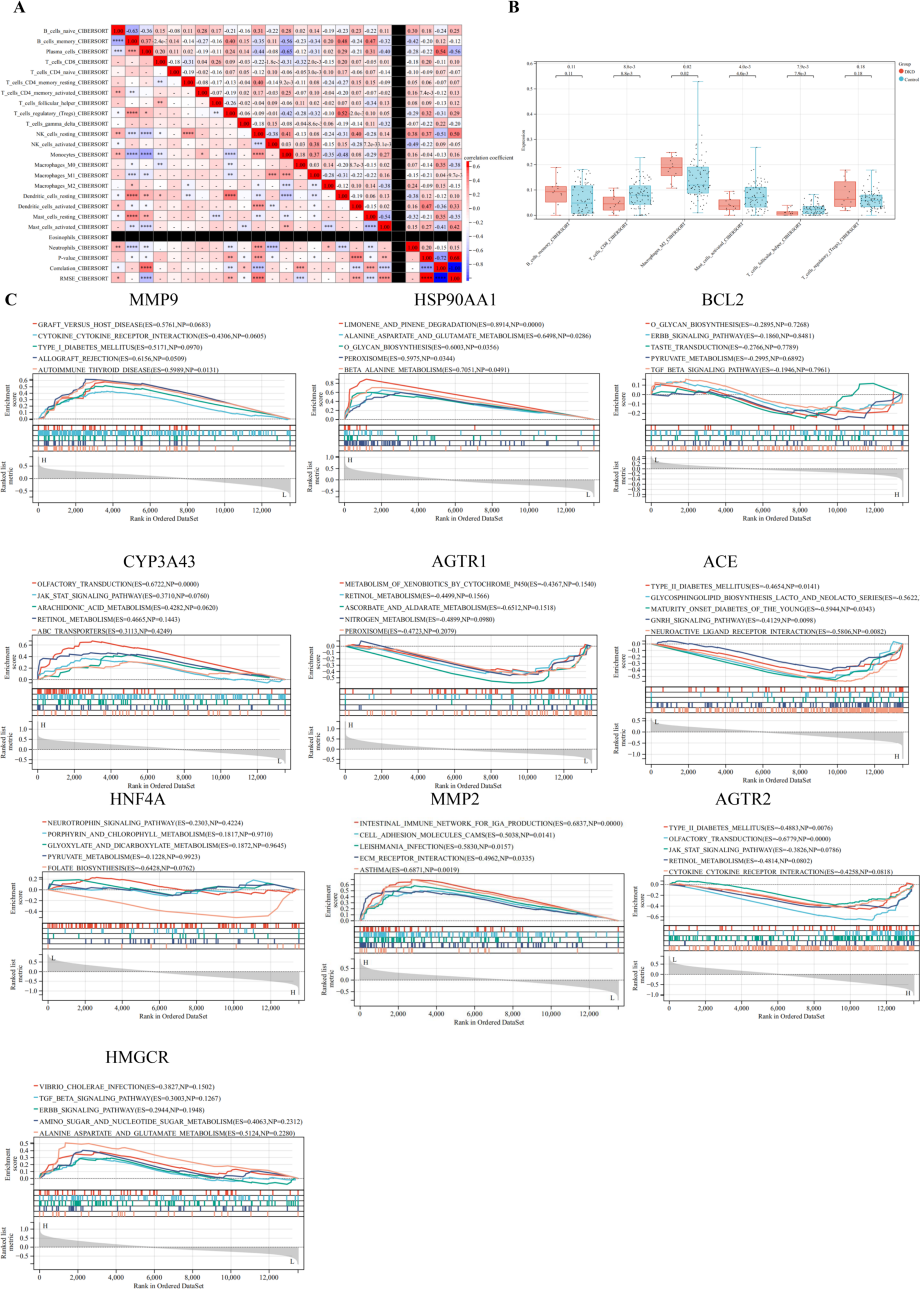
Correlation of immune cell infiltration and GSEA. **(A)** Immune infiltration correlation matrix depicting the relationships between immune cell fractions; positive correlations are indicated in red, while negative correlations are shown in blue. Only correlations with *P* < 0.05 were considered statistically significant and included in the analysis; **(B)** Boxplots depicting the differential expression of hub targets between immune cells; **(C)** GSEA of hub targets in the GSE30122 dataset. *P< 0.05,**P< 0.01,***P< 0.001,****P< 0.0001.

The immune microenvironment was characterized using CIBERSORT to assess correlations of immune cell infiltration within the GSE30122 dataset (**Figure 3A**). Moderate correlations were observed between plasma cells and resting mast cells, as well as between regulatory T cells (Tregs) and resting dendritic cells. Weak correlations were detected between activated natural killer (NK) cells and M1 macrophages, and between monocytes and M1 macrophages. Additionally, CD8+ T cells, activated mast cells, and follicular helper T cells were significantly decreased in DKD compared to controls (*P* < 0.05) (**Figure 3B**).

GSEA was performed on hub targets to elucidate potential pathogenic mechanisms. MMP9 was predominantly enriched in the allograft rejection pathway, while HSP90AA1 was associated with limonene and pinene degradation. BCL2 showed significant enrichment in the TGF-β signaling pathway. High CYP3A43 expression correlated with enrichment in olfactory transduction, whereas AGTR1 was primarily enriched in nitrogen metabolism. Elevated ACE expression was linked to maturity onset diabetes of the young, and HNF4A was enriched in pyruvate metabolism. MMP2 was associated with extracellular matrix (ECM) receptor interaction, and high AGTR2 expression correlated with Type II diabetes mellitus pathways. Finally, HMGCR was enriched in alanine, aspartate, and glutamate metabolism. Collectively, these findings suggest that these hub targets may play critical roles in immune-mediated renal injury (**Figure 3C**).

### 3.6 Molecular docking

**Figure 4:**
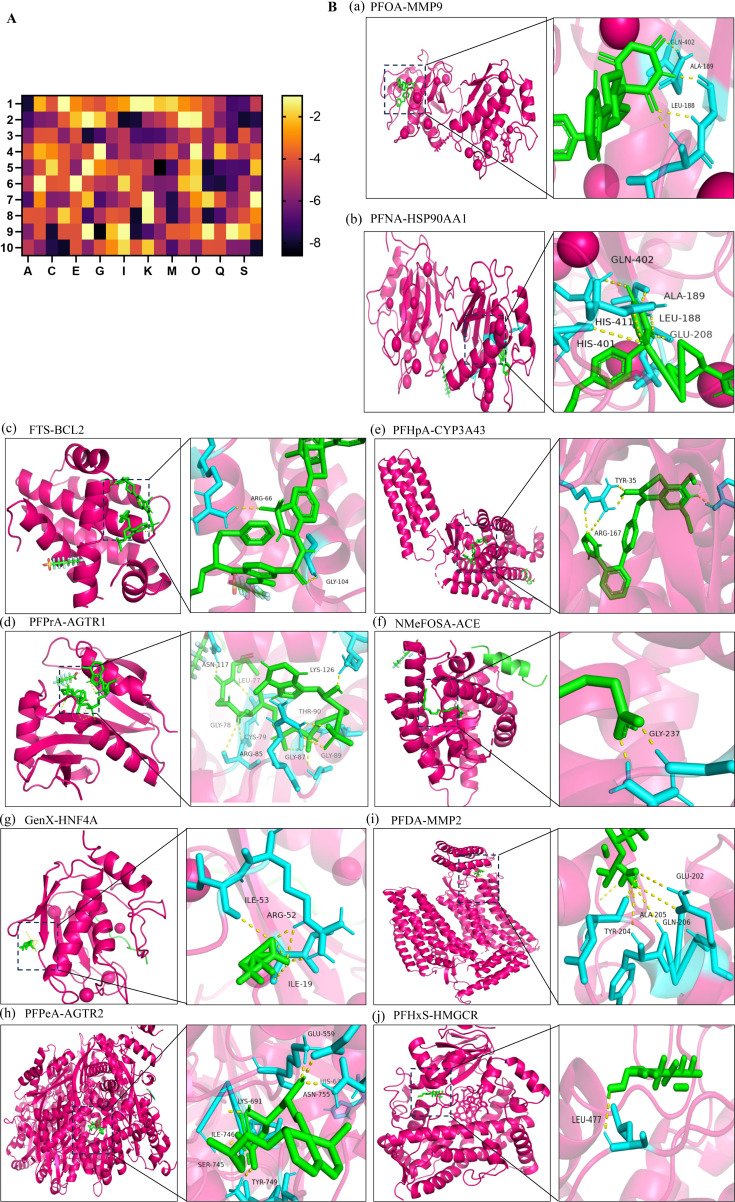
Molecular docking. **(A)** Heatmap of molecular docking between 20 types of PFAS and the top 10 hub targets; 1: MMP9 (PDB ID: 2OVX), 2: HSP90AA1 (PDB ID: 2QF6), 3: BCL2 (PDB ID: 4IEH), 4: CYP3A43 (PDB ID: 4NY4), 5: AGTR1 (PDB ID: 4YAY), 6: ACE (PDB ID: 4X5K), 7: HNF4A (PDB ID: 1PZL), 8: MMP2 (PDB ID: 3AYU), 9: AGTR2 (PDB ID: 5UNF), 10: HMGCR (PDB ID: 1HW8). A: PFOA, C: PFNA, E: FTS, G: PFPrA, I: PFHpA, K: NMeFOSA, M: GenX, O: PFPeA, Q: PFDA, S: PFHxS. **(B)** Molecular docking results with the lowest binding energy were selected for visualization using PyMOL; a: PFOA-MMP9; b: PFNA-HSP90AA1; c: FTS-BCL2; d: PFPrA-AGTR1; e: PFHpA-CYP3A43; f: NMeFOSA-ACE; g: GenX-HNF4A; h: PFPeA-AGTR2; i: PFDA-MMP2; j: PFHxS-HMGCR.

Molecular docking heatmap analysis demonstrated strong binding affinities between PFAS compounds and the top 10 hub targets (**Figure 4A**), including MMP9 (PDB ID: 2OVX), HSP90AA1 (PDB ID: 2QF6), BCL2 (PDB ID: 4IEH), CYP3A43 (PDB ID: 4NY4), AGTR1 (PDB ID: 4YAY), ACE (PDB ID: 4X5K), HNF4A (PDB ID: 1PZL), MMP2 (PDB ID: 3AYU), AGTR2 (PDB ID: 5UNF), and HMGCR (PDB ID: 1HW8). The top ten docking poses with the lowest binding energies were selected for visualization using PyMOL (40). Notably, perfluorooctanoic acid (PFOA) interacted with MMP9 residues GLN-402, ALA-189, and LEU-188, while perfluorononanoic acid (PFNA) engaged with HSP90AA1 residues GLN-402, ALA-189, LEU-188, GLU-208, HIS-411, and HIS-401. FTS bound to BCL2 at ARG-66 and GLY-104 residues, and perfluoropropanoic acid (PFPrA) interacted with AGTR1 residues ASN-117, LEU-77, LYS-126, THR-90, GLY-78, CYS-79, ARG-85, GLY-87, and GLY-89. Perfluoroheptanoic acid (PFHpA) bound CYP3A43 at TYR-35 and ARG-167 residues. The interaction between NMeFOSA and ACE involved GLY-237, whereas GenX engaged HNF4A at ILE-53, ARG-52, and ILE-19 residues. Perfluoropentanoic acid (PFPeA) bound AGTR2 at GLU-559, HIS-63, ASN-755, LYS-691, ILE-746, SER-745, and TYR-749 residues. Perfluorodecanoic acid (PFDA) interacted with MMP2 residues GLU-202, ALA-205, GLN-206, and TYR-204, and perfluorohexane sulfonate (PFHxS) bound HMGCR at LEU-477 (**Figure 4B**).

## 4 Discussion

PFAS toxicity was initially evaluated using data from PubChem, ProTox 3.0, and ChEMBL databases. Relevant PFAS targets were predicted via SwissTargetPrediction and SuperPred tools, while DKD-associated gene targets were retrieved from GeneCards and OMIM databases. The intersection of PFAS and DKD targets was used to construct a PPI network. Functional enrichment analyses, including GO and KEGG, were performed using the DAVID platform. Molecular docking simulations between PFAS compounds and hub targets were conducted utilizing Discovery Studio and the CDOCKER algorithm. Expression and localization of hub targets were validated through immunohistochemical staining, single-cell transcriptomics, and subcellular localization analyses using the HPA database, with further confirmation of gene expression in GEO datasets. Additionally, immune cell infiltration correlations and GSEA were performed to investigate potential mechanistic pathways.

PFAS are ubiquitous environmental contaminants derived from diverse industrial processes and consumer products, posing substantial risks to human health and ecological systems (41). Sources of PFAS contamination include industrial effluents, packaging materials, and manufacturing processes, with these compounds capable of bioaccumulating in plants and entering the food chain (42). Elevated PFAS levels in aquatic environments constitute a significant health hazard through water and dietary exposure. Human exposure to PFAS is widespread, raising concerns about associated health risks. Previous studies have demonstrated that PFAS can induce neurotoxicity by disrupting calcium homeostasis and altering neurotransmitter levels in neuronal cells (43). Although regulatory measures have been implemented to control the most toxic PFAS, their environmental persistence, bioaccumulative properties, and capacity to cross physiological barriers such as the blood-brain and placental barriers continue to pose long-term health risks for current and future generations. This study seeks to elucidate the mechanisms by which PFAS contribute to the development of DKD.

MMP9, BCL2, CYP3A43, ACE, HNF4A, HSP90AA1, AGTR1, MMP2, AGTR2, and HMGCR were identified as key hub targets implicated in PFAS-induced DKD toxicity. MMP9 is predominantly synthesized and secreted by glomerular mesangial cells and renal tubular epithelial cells. Reduced serum levels of MMP9 may reflect alterations in the composition of the glomerular basement membrane (GBM), particularly changes in type IV collagen turnover. Such dysregulation is characterized by increased synthesis and decreased degradation of type IV collagen, contributing to the progression of glomerulosclerosis (44). BCL2 is a prototypical anti-apoptotic gene whose primary function is to inhibit changes in mitochondrial outer membrane permeability, thereby preventing the release of cytochrome C and blocking the activation of the intrinsic apoptotic pathway. In DKD, the high-glucose and high–advanced glycation end product (AGE) microenvironment often induces apoptosis in glomerular mesangial cells, podocytes, and renal tubular epithelial cells. Downregulation of BCL2 expression is a key contributing factor in this apoptotic process (45). CYP3A43 is involved in the metabolism of steroid hormones, including testosterone, estrogen, and cortisol (46). The kidney functions as a hormone-sensitive organ, where disruptions in hormonal homeostasis can profoundly impact glomerular filtration and tubular reabsorption. ACE plays a pivotal role in the renin-angiotensin-aldosterone system (RAAS) by catalyzing the conversion of angiotensin I to angiotensin II (Ang II). Ang II mediates potent vasoconstrictive, pro-oxidative, pro-inflammatory, and pro-fibrotic effects, acting as a central driver of glomerular hypertension, mesangial cell proliferation, glomerular basement membrane thickening, and tubulointerstitial fibrosis in DKD (47). Under hyperglycemic conditions, the RAAS becomes hyperactivated, resulting in elevated expression and enzymatic activity of ACE. This exacerbates glomerular capillary hypertension, induces podocyte injury, and aggravates proteinuria. HNF4A is a critical transcriptional regulator involved in glucose metabolism and pancreatic β-cell function, and mutations in HNF4A are closely linked to maturity-onset diabetes of the young type 1 (MODY1). Aberrant expression of HNF4A within the kidney disrupts tubular metabolic homeostasis, leading to lipid accumulation, metabolic derangements, and worsening tubulointerstitial injury and fibrosis—hallmarks of both early and progressive stages of DKD (48). HSP90AA1 functions as a molecular chaperone that stabilizes crucial proteins involved in pro-inflammatory signaling pathways, including NF-κB, STAT3, and TLR4, all of which are activated in DKD. Under conditions of high glucose and AGE stimulation, HSP90AA1 expression is upregulated, thereby amplifying inflammatory responses in macrophages and renal tubular epithelial cells. This enhanced inflammation contributes to local tissue injury and promotes fibrotic progression within the kidney (49). AGTR1, a key receptor in RAAS, contributes to the progression of DKD by promoting vasoconstriction, inflammation, oxidative stress, and tissue fibrosis (50). Aberrant activation of AGTR1 in DKD is a major driver of proteinuria and glomerulosclerosis. As such, AGTR1 represents one of the central therapeutic targets in current clinical strategies aimed at mitigating DKD progression (51). MMP2, a matrix metalloproteinase involved in ECM degradation, exerts a dual regulatory role in DKD by mediating basement membrane remodeling, interstitial fibrosis, and inflammatory responses. Dysregulation of MMP2 expression disrupts ECM homeostasis, resulting in structural damage and functional impairment of renal tissue. Consequently, MMP2 is recognized as a critical pathological mediator driving the progression of DKD (52). AGTR2 may exert renoprotective effects in DKD by suppressing inflammatory responses, alleviating glomerular hyperperfusion, and attenuating tissue fibrosis (53). Excessive cholesterol synthesis and accumulation mediated by HMGCR disrupt renal lipid metabolism, thereby exacerbating oxidative stress and inflammatory responses that contribute to structural damage and fibrotic progression in both the glomeruli and renal tubules. Moreover, statins, through inhibition of HMGCR activity, effectively reduce systemic lipid levels and exert renoprotective effects that mitigate the progression of DKD. These observations underscore the pivotal role of HMGCR in the pathogenesis of DKD (54).

KEGG pathway enrichment analysis revealed that the potential toxicity of PFAS in the pathogenesis of DKD is closely associated with several key biological pathways, including nitrogen metabolism, PPAR signaling, endocrine resistance, insulin resistance, and AMPK signaling pathways. Notably, nitrogen metabolism plays a critical role in the pathophysiology of DKD, influencing renal function and metabolic homeostasis (55). Dysregulation of nitrogenous waste products, such as urea and ammonia, arises from impaired renal clearance and contributes to renal tubular toxicity and interstitial fibrosis. Elevated levels of asymmetric dimethylarginine (ADMA), an endogenous inhibitor of nitric oxide synthase, have been implicated in endothelial dysfunction and the progression of DKD by disrupting nitric oxide-mediated vasodilation and promoting oxidative stress. Furthermore, aberrant amino acid metabolism in DKD exacerbates inflammatory responses and oxidative damage, accelerating glomerulosclerosis and tubular injury. PPAR signaling pathway plays a crucial role in the pathogenesis of DKD by regulating lipid metabolism, inflammation, and fibrosis within the kidney. Among the PPAR isoforms, PPAR-γ activation has been shown to improve insulin sensitivity, reduce renal inflammation, and inhibit profibrotic signaling, thereby attenuating glomerulosclerosis and tubulointerstitial fibrosis in DKD (56). Activation of MAPK family members-including ERK1/2, JNK, and p38 MAPK-has been demonstrated to promote mesangial cell proliferation, ECM accumulation, and pro-inflammatory cytokine production, collectively contributing to glomerulosclerosis and tubulointerstitial fibrosis in DKD (57). Persistent MAPK activation under diabetic conditions leads to increased TGF-β1 expression and downstream Smad signaling, exacerbating renal fibrosis.

The identified hub targets modulate renal inflammation and fibrosis, representing promising immunotherapeutic candidates in DKD. Hub targets-including MMP2, MMP9, BCL2, ACE, AGTR1, AGTR2, HSP90AA1, HMGCR, HNF4A, and CYP3A43-are implicated in immune dysregulation associated with DKD. Specifically, MMP2 and MMP9 facilitate extracellular matrix remodeling and promote immune cell infiltration, while BCL2 plays a critical role in regulating apoptosis and maintaining immune cell survival. ACE and AGTR1/2 mediate inflammatory responses via the renin-angiotensin system (58). HSP90AA1 and HMGCR contribute to oxidative stress and pro-inflammatory signaling, while HNF4A and CYP3A43 affect immune-metabolic pathways.

The identification of toxicological targets of PFAS in the context of DKD provides novel insights into environmental contributors to renal injury. PFAS exposure has been linked to oxidative stress, inflammation, disruption of lipid metabolism, and endothelial dysfunction-pathogenic processes that accelerate DKD progression. Elucidating the molecular interactions between PFAS and critical renal pathways, including those mediated by PPAR, MAPK, and immune regulators, enhances our understanding of how environmental pollutants exacerbate DKD. Clinically, these findings underscore the importance of incorporating environmental exposure assessments into DKD risk stratification, foster the development of biomarkers for PFAS-induced nephrotoxicity, and open new avenues for preventive strategies and therapeutic interventions targeting environmentally mediated kidney damage.

In summary, this study establishes a theoretical foundation for developing treatment strategies that address the intricate relationship between PFAS exposure and DKD pathogenesis. By adopting a multifaceted approach-encompassing targeted therapies, early interventions, and vaccine development-we can effectively slow DKD progression and expand preventive and therapeutic options. Such advancements hold promise for enhancing public health initiatives and strengthening global efforts in diabetes prevention and control.

## 5 Conclusion

This study integrates network toxicology, molecular docking, and bioinformatics to advance the understanding of PFAS-related toxicity. It provides novel insights into the pathogenic mechanisms by which PFAS contribute to the development of DKD. Future therapeutic strategies targeting PFAS-such as agents designed to eliminate these compounds or inhibit their deleterious effects-may offer innovative approaches for the prevention and treatment of DKD.

## Data Availability

The original contributions presented in the study are included in the article/**Supplementary Material**, further inquiries can be directed to the corresponding author/s.
